# Changes in Urologic Cancer Surgical Volume and Length of Stay During the COVID-19 Pandemic in Pennsylvania

**DOI:** 10.1001/jamanetworkopen.2023.9848

**Published:** 2023-04-25

**Authors:** Brian Chun, Haleh Ramian, Cameron Jones, Robin Vasan, Jonathan G. Yabes, Benjamin J. Davies, Lindsay M. Sabik, Bruce L. Jacobs

**Affiliations:** 1 Department of Urology, University of Pittsburgh Medical Center, Pittsburgh, Pennsylvania; 2 Department of Health Policy and Management, University of Pittsburgh School of Public Health, Pittsburgh, Pennsylvania; 3 Urology Health Services Research Division, University of Pittsburgh School of Medicine, Pittsburgh, Pennsylvania

## Abstract

**Importance:**

Disruptions in cancer surgery during the COVID-19 pandemic led to widespread deferrals and cancellations, creating a surgical backlog that presents a challenge for health care institutions moving into the recovery phase of the pandemic.

**Objective:**

To describe patterns in surgical volume and postoperative length of stay for major urologic cancer surgery during the COVID-19 pandemic.

**Design, Setting, and Participants:**

This cohort study identified 24 001 patients 18 years or older from the Pennsylvania Health Care Cost Containment Council database with kidney cancer, prostate cancer, or bladder cancer who received a radical nephrectomy, partial nephrectomy, radical prostatectomy, or radical cystectomy between the first quarter (Q1) of 2016 and Q2 of 2021. Postoperative length of stay and adjusted surgical volumes were compared before and during the COVID-19 pandemic.

**Main Outcomes and Measures:**

The primary outcome was adjusted surgical volume for radical and partial nephrectomy, radical prostatectomy, and radical cystectomy during the COVID-19 pandemic. The secondary outcome was postoperative length of stay.

**Results:**

A total of 24 001 patients (mean [SD] age, 63.1 [9.4] years; 3522 women [15%], 19 845 White patients [83%], 17 896 living in urban areas [75%]) received major urologic cancer surgery between Q1 of 2016 and Q2 of 2021. Of these, 4896 radical nephrectomy, 3508 partial nephrectomy, 13 327 radical prostatectomy, and 2270 radical cystectomy surgical procedures were performed. There were no statistically significant differences in patient age, sex, race, ethnicity, insurance status, urban or rural status, or Elixhauser Comorbidity Index scores between patients who received surgery before and patients who received surgery during the pandemic. For partial nephrectomy, a baseline of 168 surgeries per quarter decreased to 137 surgeries per quarter in Q2 and Q3 of 2020. For radical prostatectomy, a baseline of 644 surgeries per quarter decreased to 527 surgeries per quarter in Q2 and Q3 of 2020. However, the likelihood of receiving radical nephrectomy (odds ratio [OR], 1.00; 95% CI, 0.78-1.28), partial nephrectomy (OR, 0.99; 95% CI, 0.77-1.27), radical prostatectomy (OR, 0.85; 95% CI, 0.22-3.22), or radical cystectomy (OR, 0.69; 95% CI, 0.31-1.53) was unchanged. Length of stay for partial nephrectomy decreased from baseline by a mean of 0.7 days (95% CI, −1.2 to −0.2 days) during the pandemic.

**Conclusions and Relevance:**

This cohort study suggests that partial nephrectomy and radical prostatectomy surgical volume decreased during the peak waves of COVID-19, as did postoperative length of stay for partial nephrectomy.

## Key Points

**Question:**

Did surgical volume and hospital length of stay for major urologic cancer surgical procedures change during the COVID-19 pandemic?

**Findings:**

In this cohort study of all inpatient, outpatient, and ambulatory surgery encounters in the state of Pennsylvania between the first quarter of 2016 and second quarter of 2021, comprising 24 001 patients, the volume of lower-priority urologic cancer surgical procedures significantly decreased during peak waves of COVID-19 infection. Postoperative length of stay for partial nephrectomy decreased as well.

**Meaning:**

This study suggests that, during the peak waves of the COVID-19 pandemic, urologic cancer surgery volume was predominantly reduced for low-priority cases, with reduced hospital length of stay for some surgical procedures.

## Teaser

This cohort study describes patterns in surgical volume and postoperative length of stay for major urologic cancer surgery during the COVID-19 pandemic in Pennsylvania.

## Introduction

At the peak of the COVID-19 pandemic, health care infrastructure was widely disrupted as institutions redistributed assets and personnel to address infection outbreaks. As a consequence, many elective surgical procedures were postponed due to government lockdown measures and hospital protocols aimed to preserve medical personal protective equipment, prevent iatrogenic infection, and bolster hospital capacity.^1^ Many stakeholders worry that the widespread deferral or cancellation of elective surgery, particularly cancer surgery, may result in disease progression with associated decrements in quality of life and survival outcomes.^2,3,4,5^ Widespread cancellations may also have long-term consequences that persist into the recovery phase of the pandemic, when health care systems return to normal workflow and confront a growing backlog of elective surgical procedures.

To balance COVID-19 response efforts and timely surgical services in a resource-restricted environment, governing bodies and hospitals adopted triage protocols to prioritize elective cancer surgical procedures.^6,7^ At the clinician level, surgeons postponed nonurgent elective surgical procedures^8^ and minimized patients’ postoperative length of stay.^2,9^ In the urologic community, major surgical procedures were stratified as high or low priority to help case triaging; radical cystectomy for muscle-invasive bladder cancer was recognized as urgent, most radical prostatectomies for localized prostate cancer were considered deferrable, and radical or partial nephrectomy for kidney masses ranged from elective to urgent depending on clinical tumor stage.^3^

The degree to which these practice changes are associated with surgical volume and postoperative length of stay is yet unknown, to our knowledge. Thus, we sought to examine patterns of surgical volume and postoperative length of stay among patients receiving major urologic cancer surgery during the COVID-19 pandemic. We specifically focused on 4 common urologic cancer surgical procedures—radical nephrectomy, partial nephrectomy, radical prostatectomy, and radical cystectomy—during periods of peak COVID-19 infection using the Pennsylvania Health Care Cost Containment Council (PHC4) database, which collects mandated hospital discharge information, regardless of payer, within the state of Pennsylvania.

## Methods

This study adhered to the Strengthening the Reporting of Observational Studies in Epidemiology (STROBE) reporting guideline. Our study was deemed exempt by the international review board at the University of Pittsburgh and informed consent of study participants was waived because the PHC4 is publicly reported and contains deidentified data; thus, it was deemed to present no more than minimal risk of harm to patients.

### Data Source and Study Population

The data for this study are from the PHC4 database, which collects mandated diagnosis, procedure, discharge status, length of stay, and charge data for more than 5.2 million yearly inpatient, outpatient, and ambulatory surgery center records in the state of Pennsylvania, regardless of payer.^10^ We identified all patients aged 18 years or older who received major urologic cancer surgery (radical nephrectomy, partial nephrectomy, radical prostatectomy, or radical cystectomy) between the first quarter (Q1) of 2016 and Q2 of 2021. We included patients who had a procedure code for one of these surgical procedures with an associated *International Statistical Classification of Diseases and Related Health Problems, Tenth Revision*, cancer diagnosis code at a reporting facility (eTable 1 and eTable 2 in Supplement 1). We collected demographic data, including patient age, sex, race and ethnicity, Elixhauser Comorbidity Index score, insurance payer, and rural status. Patient race and ethnicity was classified according to the PHC4 database and was included in our study as prior studies have shown inequalities in treatment patterns and access to care for different racial and ethnic groups. The Elixhauser Comorbidity Index score is a validated set of comorbidity measures optimized for use in large administrative data sets.^11^ We determined patient urban or rural status using the US Department of Agriculture rural-urban commuting area score, a 10-point designation based on travel and shopping patterns, which is calculated at the county level.^12^

### Outcomes

The primary outcomes were quarterly surgical volume and postoperative length of stay for radical nephrectomy, partial nephrectomy, radical prostatectomy, and radical cystectomy. The baseline period before COVID-19 was defined as Q1 of 2016 through Q1 of 2020. The period during COVID-19 was defined as Q2 of 2020 through Q2 of 2021, which was the most current quarter of data available. To account for changes in the population over time, we calculated adjusted surgical volume in each quarter, where the numerator was the number of patients with a procedure code for the cancer surgery of interest along with a corresponding cancer diagnosis code and the denominator was the total number of patients per quarter with that cancer diagnosis code. Postoperative length of stay was calculated as time from inpatient registration to time of discharge.

### Statistical Analysis

We compared patient demographic characteristics for patients undergoing radical nephrectomy, partial nephrectomy, radical prostatectomy, and radical cystectomy using the *t* test for continuous variables and the χ^2^ test for categorical variables. For each surgery, we fit a separate model, with length of stay as a continuous outcome variable with the before COVID-19 and during COVID-19 periods as a dependent binary variable. To analyze changes in surgical volume rates per quarter, we modeled the number of surgical procedures adjusted per 1000 patients with a relevant cancer diagnosis code to account for changes in the number of patients within the database over time. For the length of stay analysis, we used linear regression models to compare the length of stay before and during the pandemic. All models were adjusted for patient age, race and ethnicity, sex, insurance status, and number of comorbidities. All statistical analyses were performed using Stata statistical software, version 17 (StataCorp LLC). All *P* values were from 2-sided tests, and results were deemed statistically significant at *P* < .05.

## Results

A total of 24 001 patients (mean [SD] age, 63.1 [9.4] years; 3522 women [15%], 19 845 White patients [83%], 17 896 with urban living status [75%]) received major urologic cancer surgery between Q1 of 2016 and Q2 of 2021. Of these, 4896 radical nephrectomy, 3508 partial nephrectomy, 13 327 radical prostatectomy, and 2270 radical cystectomy surgical procedures were performed. Table 1 shows the demographic information for patients undergoing each surgery before and during COVID-19, as well as the odds ratio (OR) of receiving surgery during COVID-19 adjusted to these variables. There were no differences in age, insurance status, race and ethnicity, sex, urban or rural status, or Elixhauser Comorbidity Index score before and during COVID-19 for each surgery. There was no statistical difference in the likelihood of receiving radical nephrectomy (OR, 1.00; 95% CI, 0.78-1.28), partial nephrectomy (OR, 0.99; 95% CI, 0.77-1.27), radical prostatectomy (OR, 0.85; 95% CI, 0.22-3.22), or radical cystectomy (OR, 0.69; 95% CI, 0.31-1.53) during the COVID-19 pandemic.

**Table 1.** Demographic Characteristics of Patients Receiving Urologic Cancer Surgery and Adjusted Odds of Surgery During the COVID-19 Pandemic

Variable	Nephrectomy				Radical prostatectomy (n = 13 327)		Radical cystectomy (n = 2270)	
	Radical (n = 4896)		Partial (n = 3508)		Before COVID-19 (n = 11 035)	During COVID-19 (n = 2292)	Before COVID-19 (n = 1773)	During COVID-19 (n = 497)
	Before COVID-19 (n = 3873)	During COVID-19 (n = 1023)	Before COVID-19 (n = 2818)	During COVID-19 (n = 690)				
Age, No. (%)								
≤64 y	1913 (49)	458 (45)	1726 (61)	415 (60)	6843 (62)	1248 (54)	498 (28)	130 (26)
65-74 y	1162 (30)	351 (34)	808 (29)	197 (29)	3972 (36)	981 (43)	695 (39)	213 (43)
≥75 y	798 (21)	214 (21)	284 (10)	78 (11)	220 (2)	63 (3)	580 (33)	154 (31)
Insurance, No. (%)								
Private	1449 (37)	354 (34)	1302 (46)	>333 (49)^a^	6712 (61)	1299 (57)	452 (26)	134 (27)
Medicare	2013 (52)	568 (56)	1125 (40)	261 (38)	3547 (32)	822 (36)	1177 (66)	>332 (67)^a^
Medicaid	333 (9)	87 (9)	350 (12)	85 (12)	581 (5)	120 (5)	125 (7)	20 (4)
Other^b^	78 (2)	14 (1)	41 (2)	<11 (1)^a^	195 (2)	51 (2)	19 (1)	<11 (2)^a^
Race, No. (%)								
Black	353 (9)	104 (10)	376 (13)	80 (11)	1325 (12)	304 (13)	106 (6)	26 (5)
White	3283 (85)	837 (82)	2247 (80)	572 (83)	9023 (82)	1846 (81)	1585 (89)	452 (91)
Other^c^	237 (6)	82 (8)	195 (7)	38 (6)	687 (6)	142 (6)	82 (5)	19 (4)
Ethnicity, No. (%)								
Hispanic	95 (2)	26 (3)	78 (3)	13 (2)	235 (2)	47 (2)	19 (1)	<11 (1)^a^
Non-Hispanic	3778 (98)	997 (97)	2740 (97)	677 (98)	10800 (98)	2245 (98)	1754 (99)	>488 (99)^a^
Sex, No. (%)								
Male	2512 (65)	683 (67)	1785 (63)	438 (64)	11035 (100)	2292 (100)	1355 (76)	379 (76)
Female	1361 (35)	340 (33)	1033 (37)	252 (37)	0	0	418 (24)	118 (24)
Urban or rural residence, No. (%)								
Urban	2742 (71)	713 (70)	2111 (75)	524 (76)	8458 (77)	1751 (76)	1253 (71)	344 (69)
Rural	1131 (29)	310 (30)	707 (25)	166 (24)	2577 (23)	541 (24)	520 (29)	153 (31)
Elixhauser Comorbidity Index score, No. (%)								
<5	2334 (60)	609 (60)	2211 (79)	527 (76)	9641 (87)	1922 (84)	751 (42)	188 (38)
5-13	866 (22)	236 (23)	412 (15)	115 (17)	1091 (10)	275 (12)	486 (27)	126 (25)
>13	673 (17)	178 (17)	195 (7)	48 (7)	303 (3)	95 (4)	536 (30)	183 (37)
Odds ratio, (95% CI)^d^	1 [Reference]	1.00 (0.78-1.28)	1 [Reference]	0.99 (0.77-1.27)	1 [Reference]	0.85 (0.22-3.22)	1 [Reference]	0.69 (0.31-1.53)

^a^ Pennsylvania Health Care Cost Containment Council data reporting and privacy guidelines prohibit reporting discrete values for cells with fewer than 11 patients. For data where low values must be masked, guidelines also require masking the highest value for that particular cell.

^b^ Classified as other than private, Medicare, or Medicaid, or not reported.

^c^ Classified as other than White or Black, or not reported.

^d^ Adjusted for age, insurance status, race and ethnicity, sex, urban or rural residence, and Elixhauser Comorbidity Index score.

**Figure 1.**
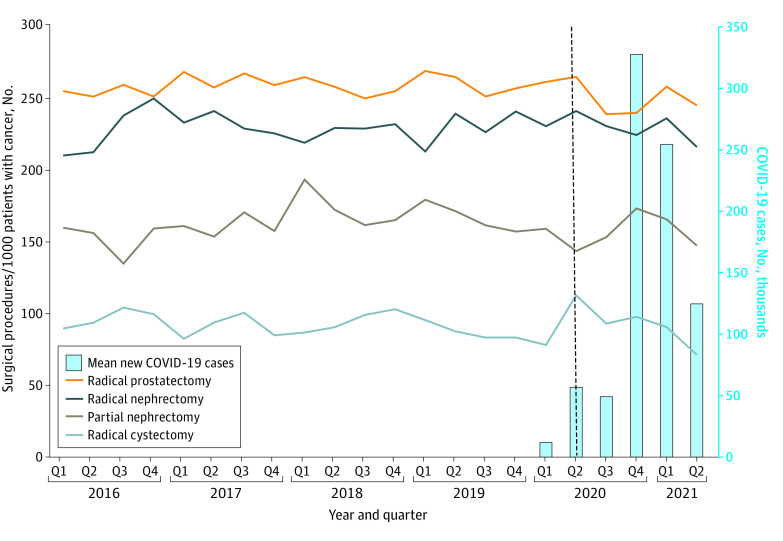
Quarterly Volume of Surgical Procedures Performed per Surgery Adjusted to 1000 Patients With Diagnosis of Each Cancer Dotted vertical line indicates the beginning of the COVID-19 pandemic. Partial nephrectomy and radical prostatectomy rates decreased during quarter 2 (Q2) and quarter 3 (Q3) of 2020. Radical cystectomy rates during the same period increased. Radical nephrectomy rates were unchanged during the COVID-19 pandemic.

There were decreases in volume for some surgical procedures when stratified by quarter (Figure 1). Although radical nephrectomy rates remained unchanged throughout the COVID-19 period compared with baseline volumes, partial nephrectomy and radical prostatectomy rates had the greatest decreases from baseline during Q2 and Q3 of 2020, corresponding to the first waves of the COVID-19 pandemic. For partial nephrectomy, a baseline of 168 surgeries per quarter decreased to 137 surgeries per quarter in Q2 and Q3 of 2020. For radical prostatectomy, a baseline of 644 surgeries per quarter decreased to 527 surgeries per quarter in Q2 and Q3 of 2020. In contrast, radical cystectomy rates during the same period increased before returning to baseline levels in Q4 of 2020 (eFigure 1A-D in Supplement 1).

**Figure 2.**
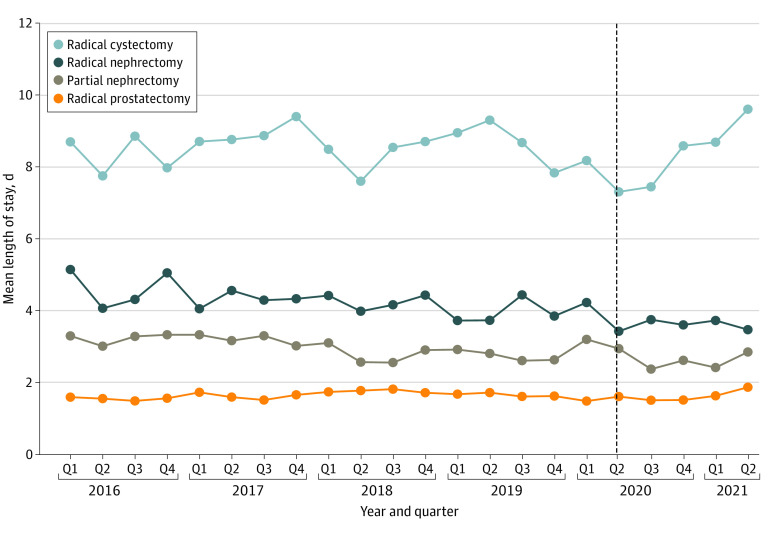
Mean Postoperative Length of Stay for Urologic Cancer Surgical Procedures by Quarter Dotted vertical line indicates the beginning of the COVID-19 pandemic. Partial nephrectomy length of stay decreased a mean of 0.7 days during the COVID-19 pandemic. Length of stay for radical nephrectomy, radical prostatectomy, and radical cystectomy did not change during the pandemic.

Figure 2 presents quarterly trends in mean postoperative length of stay for each surgery before and during the COVID-19 pandemic. There were no changes in adjusted length of stay for radical nephrectomy (−0.6 days; 95% CI, −1.3 to 0.2 days), radical prostatectomy (0.04 days; 95% CI, −0.2 to 0.3 days), or radical cystectomy (−0.5 days; 95% CI, −1.9 to 1.0 days) (Table 2; eFigure 2A-D in Supplement 1). Partial nephrectomy length of stay decreased by an adjusted mean of 0.7 days (95% CI, −1.2 to −0.2 days) during the pandemic compared with baseline (Table 2).

**Table 2.** Change in Postoperative Length of Stay After Major Urologic Cancer Surgery

Variable	Odds ratio (95% CI)			
	Radical nephrectomy	Partial nephrectomy	Radical prostatectomy	Radical cystectomy
Adjusted difference, d^a^	−0.6 (−1.3 to 0.2)	−0.7 (−1.2 to −0.2)	0.04 (−0.2 to 0.3)	−0.5 (−1.9 to 1.0)
Approach				
Minimally invasive	1 [Reference]	1 [Reference]	1 [Reference]	1 [Reference]
Open surgery	1.47 (1.19 to 1.74)	1.34 (1.16 to 1.52)	1.12 (0.98 to 1.27)	1.16 (0.55 to 1.78)
Age				
<65 y	1 [Reference]	1 [Reference]	1 [Reference]	1 [Reference]
65-74 y	−0.05 (−0.47 to 0.37)	0.01 (−0.24 to 0.27)	0.03 (−0.10 to 0.15)	0.63 (−0.21 to 1.47)
≥75 y	0.23 (−0.24 to 0.71)	0.66 (0.31 to 1.00)	1.34 (1.04 to 1.64)	0.85 (−0.05 to 1.75)
Insurance				
Private	1 [Reference]	1 [Reference]	1 [Reference]	1 [Reference]
Medicare	0.47 (0.06 to 0.88)	0.43 (0.18 to 0.69)	0.17 (0.03 to 0.30)	−0.02 (−0.80 to 0.76)
Medicaid	0.69 (0.17 to 1.21)	0.34 (0.06 to 0.61)	0.22 (0 to 0.43)	1.82 (0.67 to 2.96)
Other^b^	−0.18 (−1.18 to 0.83)	0.11 (−0.63 to 0.84)	0.20 (−0.14 to 0.53)	0.54 (−1.79 to 2.87)
Race				
White	1 [Reference]	1 [Reference]	1 [Reference]	1 [Reference]
Black	0.89 (0.41 to 1.37)	0.14 (−0.12 to 0.40)	0.27 (0.12 to 0.41)	1.46 (0.36 to 2.55)
Other^c^	0.50 (−0.19 to 1.18)	−0.08 (−0.51 to 0.34)	0.01 (−0.22 to 0.25)	0.25 (−1.11 to 1.62)
Ethnicity				
Non-Hispanic	1 [Reference]	1 [Reference]	1 [Reference]	1 [Reference]
Hispanic	−1.24 (−2.33 to −0.15)	0.12 (−0.55 to 0.79)	−0.09 (−0.52 to 0.33)	0.60 (−2.23 to 3.44)
Sex				
Female	1 [Reference]	1 [Reference]	NA	1 [Reference]
Male	−0.08 (−0.36 to 0.21)	−0.07 (−0.24 to 0.10)	NA	−0.11 (−0.71 to 0.49)
Rural or urban status				
Urban	1 [Reference]	1 [Reference]	1 [Reference]	1 [Reference]
Rural	−0.39 (−0.70 to −0.09)	−0.16 (−0.36 to 0.04)	−0.07 (−0.19 to 0.05)	−0.87 (−1.43 to −0.31)
Elixhauser Comorbidity Index score				
<5	1 [Reference]	1 [Reference]	1 [Reference]	1 [Reference]
5-13	1.21 (0.88 to 1.55)	1.07 (0.84 to 1.30)	0.64 (0.50 to 0.78)	1.81 (1.18 to 2.43)
>13	4.32 (3.95 to 4.69)	3.00 (2.68 to 3.32)	2.83 (2.59 to 3.06)	4.86 (4.26 to 5.47)

Abbreviation: NA, not applicable.

^a^ Adjusted for age, insurance status, race, ethnicity, sex, urban or rural residence, and Elixhauser Comorbidity Index score.

^b^ Classified as other than private, Medicare, Medicaid, or not reported.

^c^ Classified as other than White, Black, or not reported.

Surgery via an open approach and higher Elixhauser Comorbidity Index score were associated with longer length of stay for all surgical procedures (Table 2). Black race was associated with longer length of stay after radical nephrectomy (OR, 0.89; 95% CI, 0.41-1.37), radical prostatectomy (OR, 0.27; 95% CI, 0.12-0.41), and radical cystectomy (OR, 1.46; 95% CI, 0.36-2.55). Age older than 75 years was associated with longer length of stay after partial nephrectomy (OR, 0.66; 95% CI 0.31-1.00) and radical prostatectomy (OR, 1.34; 95% CI 1.04-1.64). Rural status was associated with shorter length of stay for radical nephrectomy (OR, −0.39; 95% CI, −0.70 to −0.09) and radical cystectomy (OR, −0.87; 95% CI, −1.43 to −0.31).

## Discussion

During the COVID-19 pandemic, the adjusted likelihood of receiving major urologic cancer surgery (radical nephrectomy, partial nephrectomy, radical prostatectomy, or radical cystectomy) did not significantly change; however, there were decreases in surgical volume during specific quarters coinciding with peaks in COVID-19 cases. In addition, postoperative length of stay after partial nephrectomy decreased by an adjusted mean of 0.7 days during the COVID-19 pandemic, while other surgical procedures showed no differences.

The most significant reductions in surgical volume were between Q2 of 2020 and Q4 of 2020, which align with the first 2 major waves of the COVID-19 pandemic. On March 19, 2020, Pennsylvania issued statewide closure of nonessential operations, and a stay-at-home order was enacted March 23, 2020. On November 30, 2020, Pennsylvania issued a state order to reduce elective surgery by 50% in regions heavily affected by the pandemic. These lockdown measures significantly reduced hospital surgical capacity as resources were diverted to COVID-19–related care and most surgical procedures were deferred to limit iatrogenic COVID-19 infection risk.^13,14^ COVID-19 had a disproportionate association with radical prostatectomy and partial nephrectomy (up to an 8% and 12% quarterly decrease, respectively). This finding aligns with the triaging consensus from the urologic community and the American College of Surgeons guidelines that considered these surgical procedures lower priority that could be safely deferred.^3^ Although quarterly rates for low-priority surgical procedures decreased significantly during peak COVID-19 infection, the overall likelihood of receiving any of these surgical procedures did not change for the entire pandemic period. We postulate that this is due to a rebound in surgical volume by Q1 of 2021 that recouped deferred surgical procedures as the pandemic entered the recovery phase. We noted a transient 5% quarterly increase in cystectomy rates in Q2 of 2020, which may suggest a shift to perform more urgent surgical procedures at the expense of low-priority cases under restricted surgical capacity.

The decrease in surgical volume during peak disruption is not only expected to have repercussions in the acute phase, but also into the recovery phase of the pandemic as a backlog of deferred surgical procedures mounts. A bayesian model of surgical volume during a 12-week COVID-19 disruption predicted that 38% of global cancer surgical procedures would be cancelled and that, even if surgical volume increased 20% above normal capacity, it would still take a median 45 weeks to clear the backlog.^15^ In addition, this disruption is more severe in middle- and high-income countries such as the United States.

Several groups have published tiered guidelines to facilitate triage of urologic cancer surgical procedures.^16^ At many institutions, multidisciplinary surgical committees were established to review scheduled surgical procedures that were then prioritized on a case-by-case basis.^17^ Central to these guidelines, there is a consensus on surgical procedures with urgent indications that should not be postponed, such as radical cystectomy for muscle-invasive bladder cancer and radical nephrectomy for cT3 kidney masses. Conversely, prostatectomy for localized intermediate- and high-risk prostate cancer and small kidney masses is predominantly considered elective and may be safely deferred or the patient placed on active surveillance.^3,4,16,18^ It is less clear how to best triage midtier or “gray-zone” surgical procedures—such as cT2 kidney masses—as the ramifications of postponing these surgical procedures are not well understood.

We did not observe significant differences in postoperative length of stay for radical nephrectomy, radical prostatectomy, or radical cystectomy during the COVID-19 pandemic. This finding suggests that these surgical procedures already have optimized inpatient hospital courses with not as much “fat to trim,” even in a pandemic setting that prioritized hospital bed capacity to address emerging COVID-19 outbreaks. Partial nephrectomy hospital length of stay decreased 0.7 days (*P* < .001) during the COVID-19 period. The reasons for this decrease are likely multifactorial, and its clinical significance is unclear. A recent survey of surgeons showed that many prioritized shortening postoperative hospital stays whenever possible to reduce the risk of iatrogenic COVID-19 infection.^8^ Moreover, many institutions have expanded same-day surgery to robotic prostatectomy and partial nephrectomy to reduce hospital bed occupancy, with early data suggesting equivalent readmission rates.^19^ Widespread adoption of Enhanced Recovery After Surgery protocols could also be used to trim length of stay and increase surgical throughput.^20^

The COVID-19 pandemic has been associated with exacerbating existing health care disparities, particularly in telehealth^21^ and cancer screening.^22,23^ Our study did not observe demographic differences between those who received surgery before and during the COVID-19 pandemic, although this study represents a selective cohort of patients with urologic malignant neoplasms and cannot be generalized to all surgical cancer care. The effect of the COVID-19 pandemic on cancer surgery continues to be unprecedented, with government, institutional, and clinician decision-making affecting the delivery of care. The challenges of the pandemic era will also persist into the recovery phase as health care systems address the expected backlog of deferred surgical procedures. Our findings quantify how surgical volume was affected by this unprecedented health crisis and restrictive government legislation and will be useful to inform protocols that redistribute health care resources in a way that balances surgical cancer care with other public health priorities. Restrictions on nonessential elective surgery can be an effective measure to divert resources in future pandemics insofar that institutions have reasonable capability to address the expected surgical backlog. We found no differences in the likelihood of receiving urologic cancer surgery during the pandemic; however, morbidity associated with delays in surgical care is less clear and requires further investigation.

### Limitations

Our study has several limitations. It is a retrospective analysis of a hospital discharge database that relies on diagnosis and hospital billing codes and does not include cancer staging information. Because surgical volume was adjusted for each 1000 patients in the database with an appropriate diagnosis code, disease prevalence may have been undercounted if patients were less likely to receive hospital-based care during the pandemic. As a result, reductions in surgical volume may be underestimated in this model. Our findings may not be generalizable to states with different COVID-19 case patterns and local government policies. Nevertheless, these findings are likely to mirror the experiences of many states with similar populations, urban or rural distribution, and lockdown restrictions. Last, our study did not account for patient factors associated with surgical volume. A survey of patients scheduled for urologic cancer surgery during the COVID-19 pandemic at 2 high-volume Italian centers found that 33% would defer surgery, with 80% of those patients willing to defer surgery beyond 6 months.^24^ Nearly 40% of patients considered risk of COVID-19 infection more harmful to their health than postponing cancer surgery.

## Conclusions

In this cohort study of patients receiving major urologic cancer surgery in Pennsylvania, the volume of lower-priority urologic cancer surgical procedures (partial nephrectomy and radical prostatectomy) significantly decreased during the peak waves of the COVID-19 pandemic. Postoperative length of stay after partial nephrectomy decreased by an adjusted mean of 0.7 days (95% CI, −1.2 to −0.2 days) during the pandemic compared with baseline but was unchanged for radical nephrectomy, radical prostatectomy, and radical cystectomy.

## Notes

Supplement 1. **eTable 1.** *International Classification of Diseases* (*ICD*)-*10* Cancer Diagnosis Codes for Urologic Cancers **eTable 2.** Procedure Codes for Radical Nephrectomy, Partial Nephrectomy, Radical Prostatectomy, Radical Cystectomy **eFigure 1.** Quarterly Volume of Surgeries Performed per 1000 Patients With Diagnosis of Each Cancer **eFigure 2.** Average Postoperative Length of Stay per Quarter by Surgery

Supplement 2.
Data Sharing Statement
