# Supplementary material for: Changes in Urologic Cancer Surgical Volume and Length of Stay During the COVID-19 Pandemic in Pennsylvania
Source: JAMA Netw Open. 2023 Apr 25;6(4):e239848. doi: 10.1001/jamanetworkopen.2023.9848 (PMC10130946; doi:10.1001/jamanetworkopen.2023.9848)
Supplement: Supplement 1. — eTable 1. International Classification of Diseases (ICD)-10 Cancer Diagnosis Codes for Urologic Cancers eTable 2. Procedure Codes for Radical Nephrectomy, Partial Nephrectomy, Radical Prostatectomy, Radical Cystectomy eFigure 1. Quarterly Volume of Surgeries Performed per 1000 Patients With Diagnosis of Each Cancer eFigure 2. Average Postoperative Length of Stay per Quarter by Surgery [file jamanetwopen-e239848-s001.pdf]

Supplementary Online Content

Chun B, Ramian H, Jones C, et al. Changes in urologic cancer surgical volume and length of stay during the COVID-19 pandemic in Pennsylvania. *JAMA Netw Open.* 2023;6(4):e239848. doi:10.1001/jamanetworkopen.2023.9848

eTable 1. *International Classification of Diseases* (*ICD*)-*10* Cancer Diagnosis Codes for Urologic Cancers
eTable 2. Procedure Codes for Radical Nephrectomy, Partial Nephrectomy, Radical Prostatectomy, Radical Cystectomy
eFigure 1. Quarterly Volume of Surgeries Performed per 1000 Patients With Diagnosis of Each Cancer
eFigure 2. Average Postoperative Length of Stay per Quarter by Surgery

This supplementary material has been provided by the authors to give readers additional information about their work.

© 2023 Chun B et al. *JAMA Network Open.*

eTable 1. *International Classification of Diseases* (*ICD*)*-10* Cancer Diagnosis Codes for Urologic Cancers

**Prostate Cancer**	
C61	Malignant neoplasm of prostate
D07.5	Carcinoma in situ of prostate
**Kidney Cancer**	
C64.1	Malignant neoplasm of right kidney, except renal pelvis
C64.2	Malignant neoplasm of left kidney, except renal pelvis
C64.9	Malignant neoplasm of unspecified kidney, except renal pelvis
**Bladder Cancer**	
C67	Malignant neoplasm of bladder
C67.0	Malignant neoplasm of trigone of bladder
C67.1	Malignant neoplasm of dome of bladder
C67.2	Malignant neoplasm of lateral wall of bladder
C67.3	Malignant neoplasm of anterior wall of bladder
C67.4	Malignant neoplasm of posterior wall of bladder
C67.5	Malignant neoplasm of bladder neck
C67.6	Malignant neoplasm of ureteric orifice
C67.7	Malignant neoplasm of urachus
C67.8	Malignant neoplasm of overlapping sites of bladder
C67.9	Malignant neoplasm of bladder, unspecified
D09.0	Carcinoma in situ of bladder

© 2023 Chun B et al. *JAMA Network Open.*

eTable 2. Procedure Codes for Radical Nephrectomy, Partial Nephrectomy, Radical Prostatectomy, Radical Cystectomy

**Radical Nephrectomy**	
0TT20ZZ	Resection of Bilateral Kidneys, Open Approach
0TT24ZZ	Resection of Bilateral Kidneys, Percutaneous Endoscopic Approach
0TT00ZZ	Resection of Right Kidney, Open Approach
0TT10ZZ	Resection of Left Kidney, Open Approach
0TT04ZZ	Resection of Right Kidney, Percutaneous Endoscopic Approach
0TT14ZZ	Resection of Left Kidney, Percutaneous Endoscopic Approach
**Partial Nephrectomy**	
0TB00ZZ	Excision of Right Kidney, Open Approach
0TB04ZZ	Excision of Right Kidney, Percutaneous Endoscopic Approach
0TB10ZZ	Excision of Left Kidney, Open Approach
0TB14ZZ	Excision of Left Kidney, Percutaneous Endoscopic Approach
**Radical Prostatectomy**	
0VT00ZZ	Resection of Prostate, Open Approach
0VT04ZZ	Resection of Prostate, Percutaneous Endoscopic Approach
0VT30ZZ	Resection of Bilateral Seminal Vesicles, Open Approach
0VT34ZZ	Resection of Bilateral Seminal Vesicles, Percutaneous Endoscopic Approach
**Radical Cystectomy**	
0TTB0ZZ	Resection of Bladder, Open Approach
0TTB4ZZ	Resection of Bladder, Percutaneous Endoscopic Approach

© 2023 Chun B et al. *JAMA Network Open.*

eFigure 1. Quarterly Volume of Surgeries Performed per 1000 Patients With Diagnosis of Each Cancer

a.

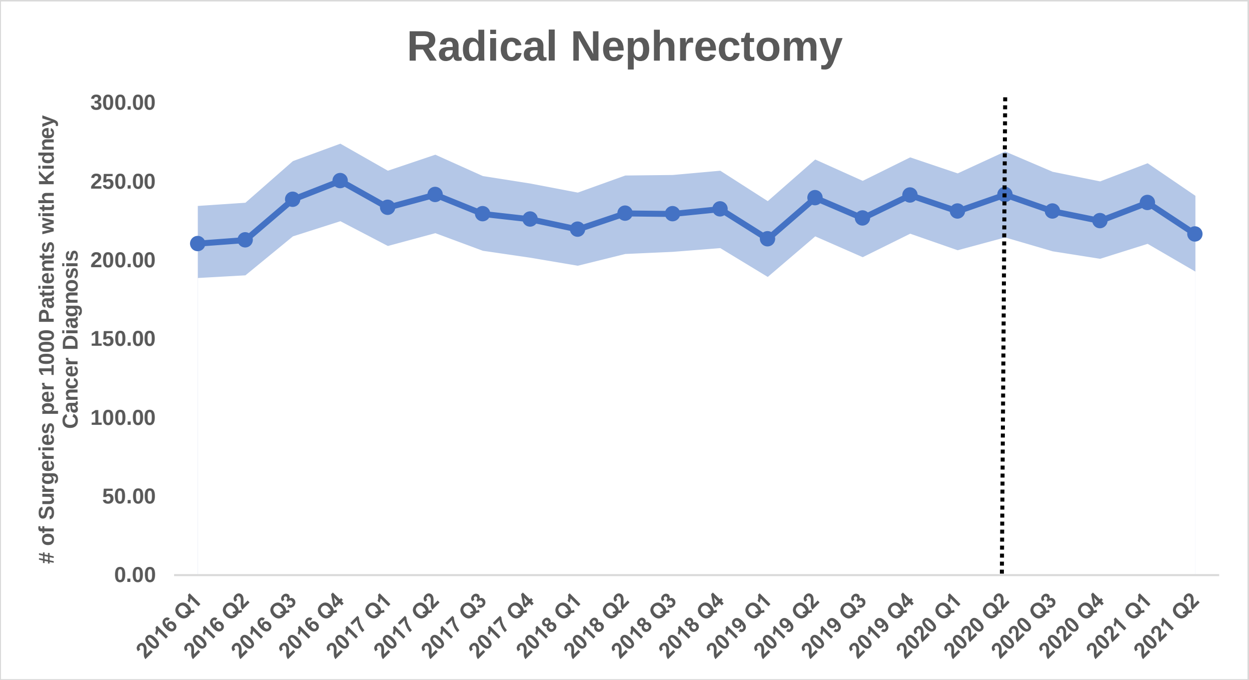

© 2023 Chun B et al. *JAMA Network Open.*

b.

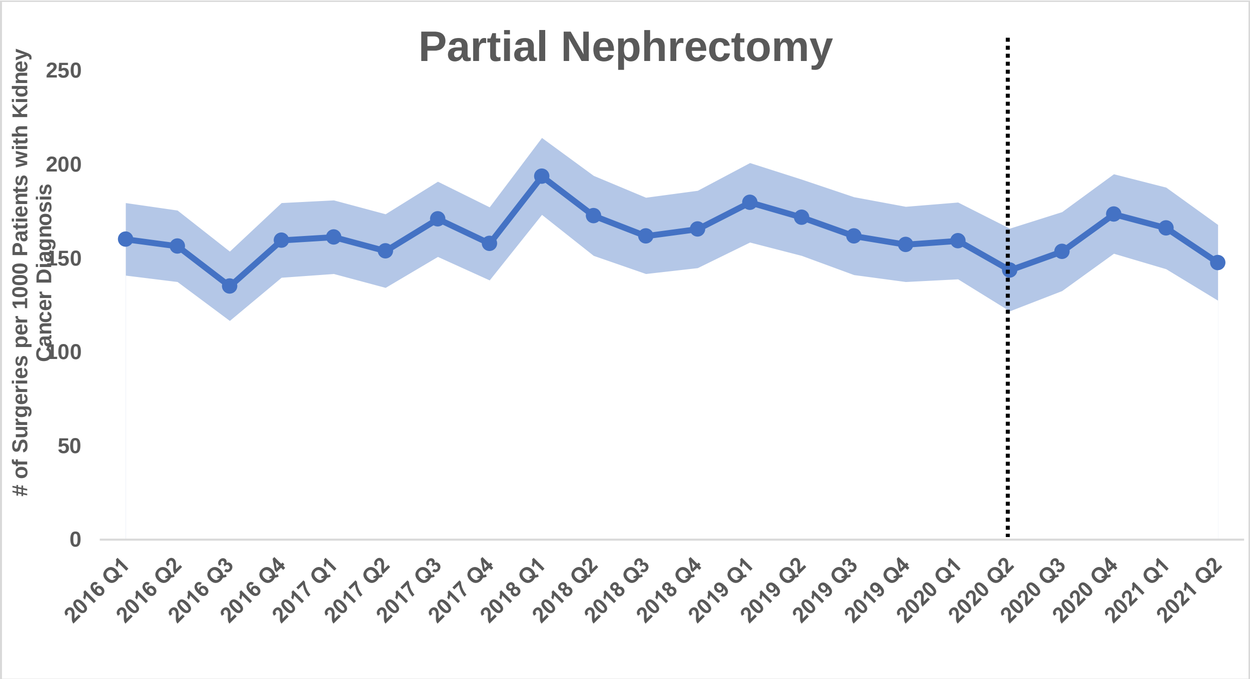

© 2023 Chun B et al. *JAMA Network Open.*

c.

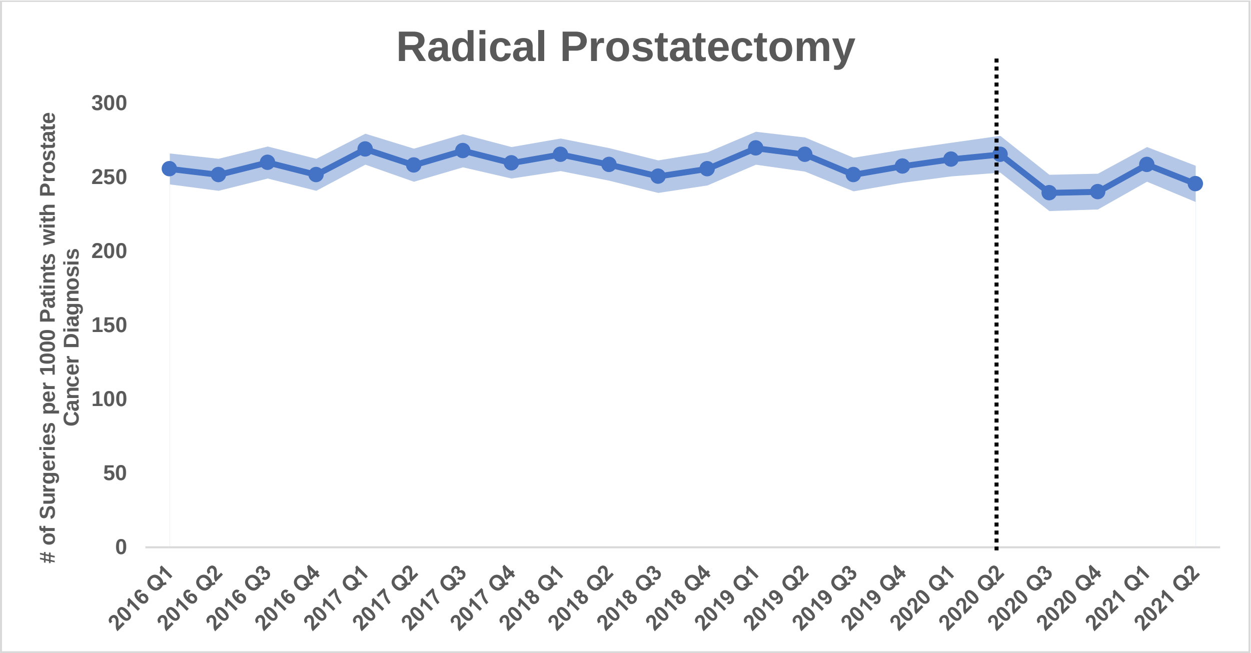

© 2023 Chun B et al. *JAMA Network Open.*

d.

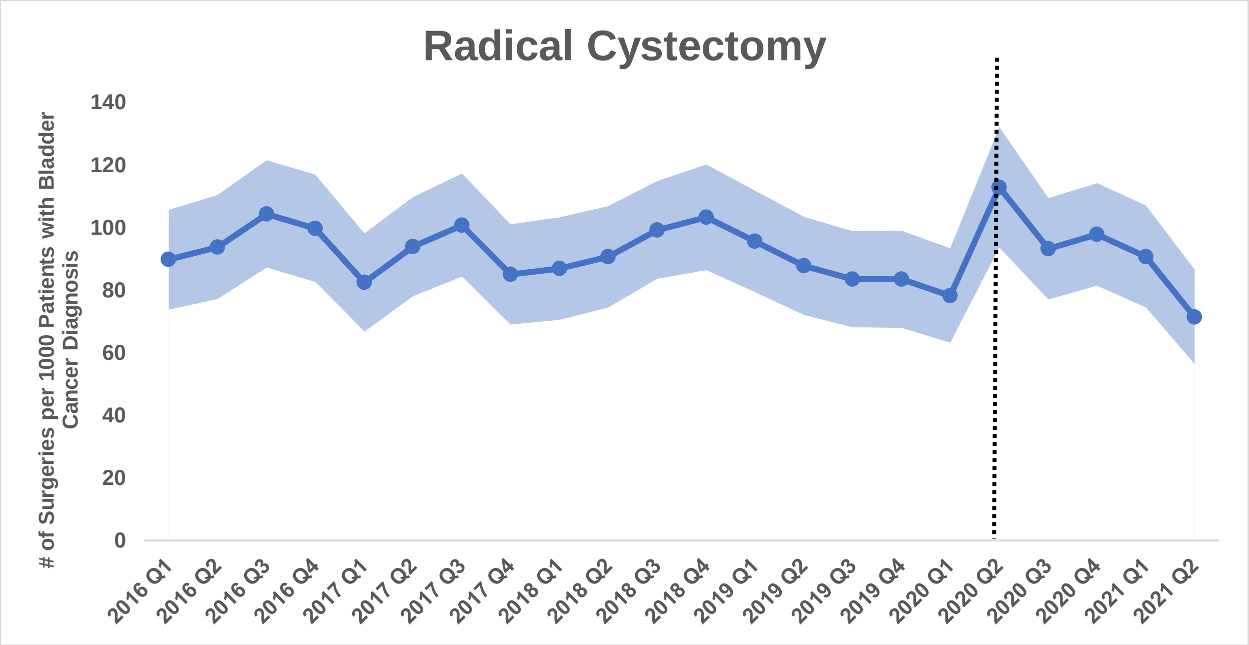

95% confidence intervals shown in shaded blue. Dotted black line represents the beginning of the COVID-19 period.

© 2023 Chun B et al. *JAMA Network Open.*

eFigure 2. Average Postoperative Length of Stay per Quarter by Surgery

a.

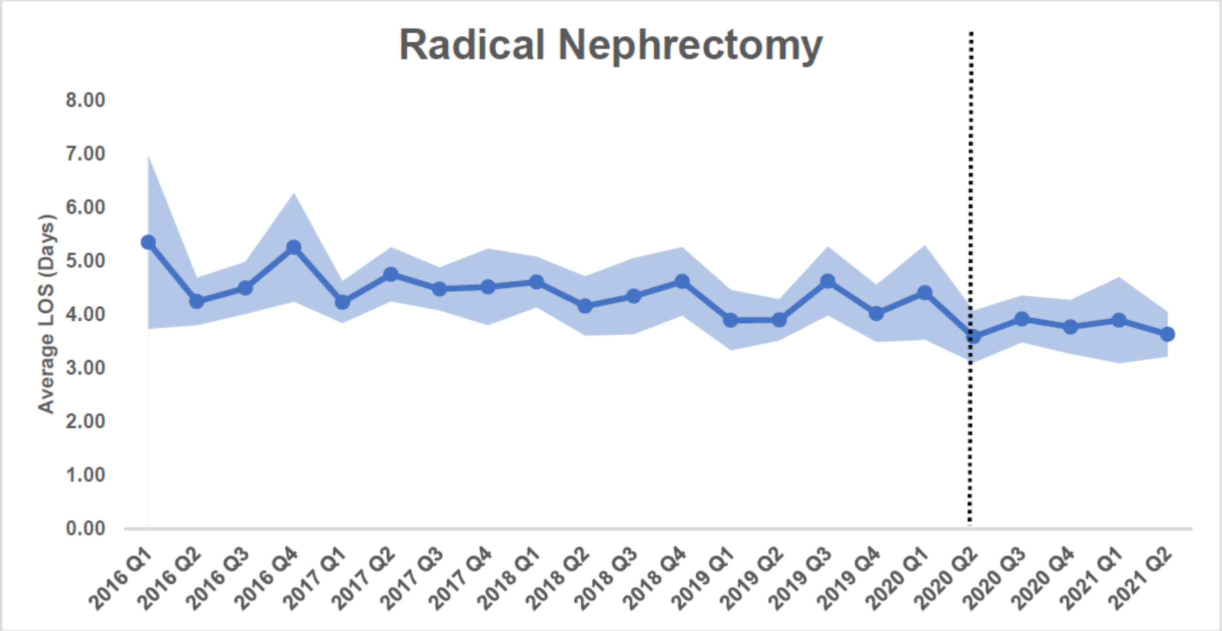

b.

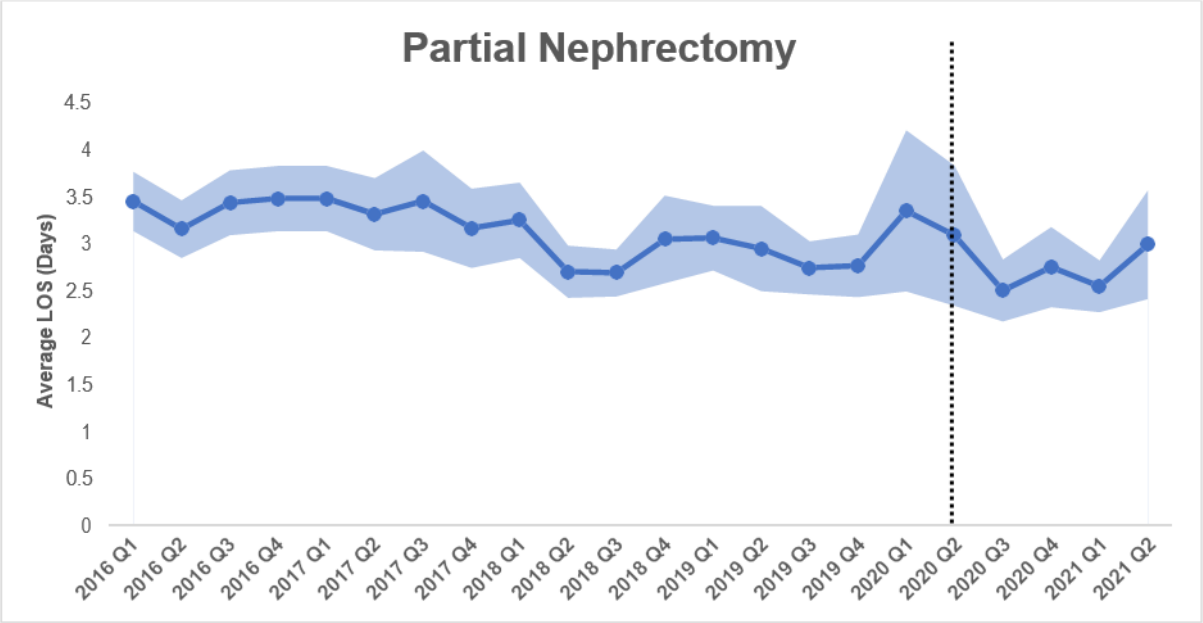

© 2023 Chun B et al. *JAMA Network Open.*

c.

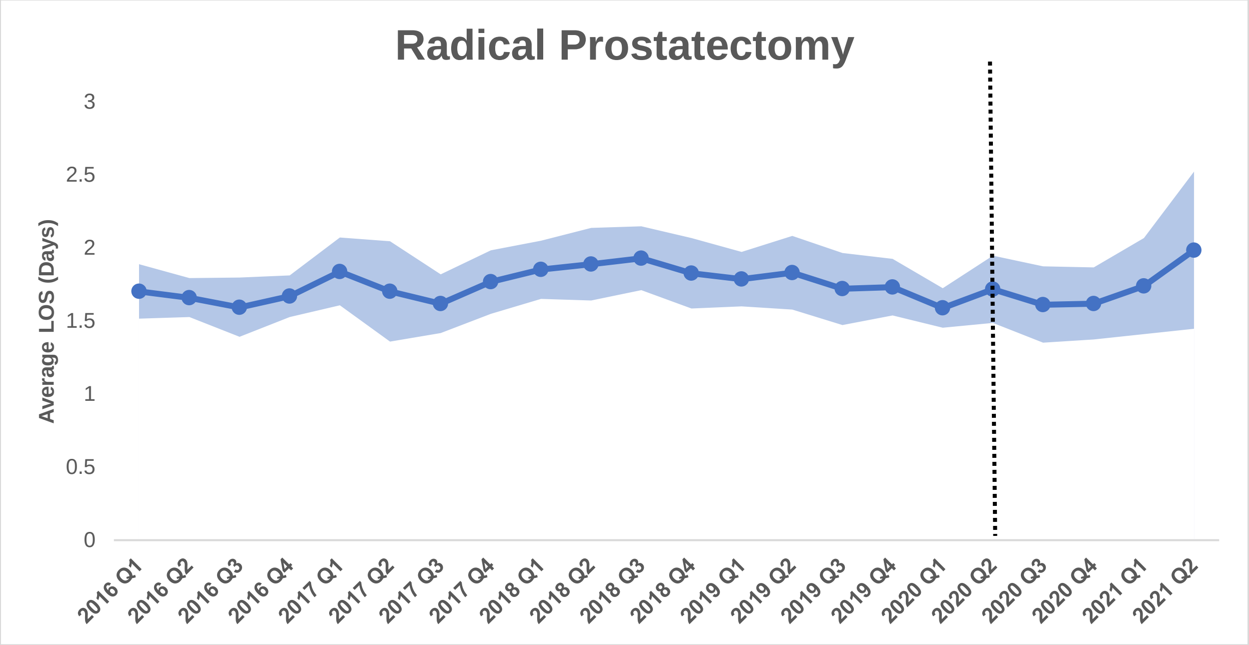

d.

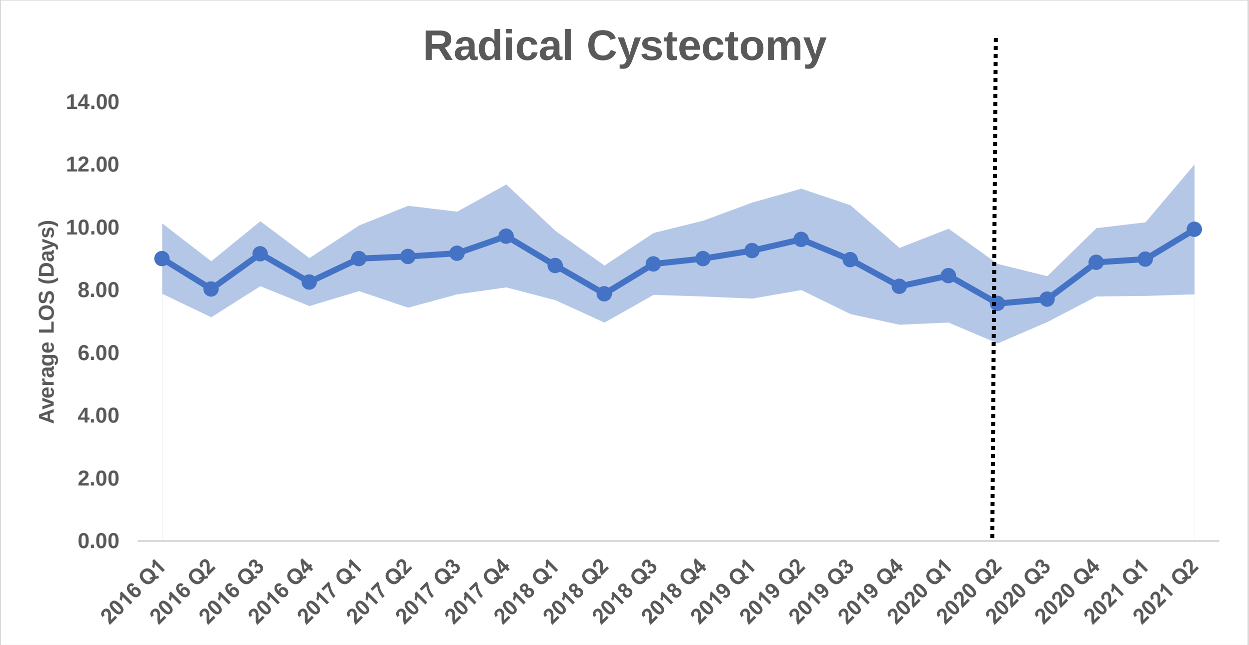

95% confidence intervals shown in shaded blue. Dotted black line represents the beginning of the COVID-19 period.

© 2023 Chun B et al. *JAMA Network Open.*
