# Supplementary material for: Changes in Urologic Cancer Surgical Volume and Length of Stay During the COVID-19 Pandemic in Pennsylvania
Source: JAMA Netw Open. 2023 Apr 25;6(4):e239848. doi: 10.1001/jamanetworkopen.2023.9848 (PMC10130946; doi:10.1001/jamanetworkopen.2023.9848)
Supplement: Supplement 2. — Data Sharing Statement [file jamanetwopen-e239848-s002.pdf]

Data Sharing Statement

Chun. Changes in Urologic Cancer Surgical Volume and Length of Stay During the COVID-19 Pandemic in Pennsylvania. *JAMA Netw Open*. Published April 25, 2023.
doi:10.1001/jamanetworkopen.2023.9848

Data
Data available: No
